# Feudalistic Platooning: Subdivide Platoons, Unite Networks, and Conquer Efficiency and Reliability

**DOI:** 10.3390/s22124484

**Published:** 2022-06-14

**Authors:** Tobias Renzler, Michael Stolz, Daniel Watzenig

**Affiliations:** 1 Institute of Automation and Control, Graz University of Technology, 8010 Graz, Austria; michael.stolz@tugraz.at (M.S.); daniel.watzenig@tugraz.at (D.W.); 2 Virtual Vehicle Research GmbH, 8010 Graz, Austria

**Keywords:** C-ITS, platooning, platoon management, IEEE 802.11p, C-V2X, Omnet++, SUMO

## Abstract

Cooperative intelligent transportation systems (C-ITSs) such as platooning rely on a robust and timely network that may not always be available in sufficient quality. Out of the box hybrid networks only partly eliminate shortcomings: mutual interference avoidance, data load balancing, and data dissemination must be sophisticated. Lacking network quality may lead to safety bottlenecks that require that the distance between the following vehicles be increased. However, increasing gaps result in efficiency loss and additionally compromise safety as the platoon is split into smaller parts by traffic: maneuvers, e.g., cut-in maneuvers bear safety risks, and consequently lower efficiency even further. However, platoons, especially if they are very long, can negatively affect the flow of traffic. This mainly applies on entry or exit lanes, on narrow lanes, or in intersection areas: automated and non-automated vehicles in traffic do affect each other and are interdependent. To account for varying network quality and enable the coexistence of non-automated and platooned traffic, we present in this paper a new concept of platooning that unites ad hoc—in form of IEEE 802.11p—and cellular communication: feudalistic platooning. Platooned vehicles are divided into smaller groups, inseparable by surrounding traffic, and are assigned roles that determine the communication flow between vehicles, other groups and platoons, and infrastructure. Critical vehicle data are redundantly sent while the ad hoc network is only used for this purpose. The remaining data are sent—relying on cellular infrastructure once it is available—directly between vehicles with or without the use of network involvement for scheduling. The presented approach was tested in simulations using Omnet++ and Simulation of Urban Mobility (SUMO).

## 1. Introduction

Platoons are convoys of several vehicles in which the first vehicle is driven manually and the following vehicles are automated. Automation within a platoon takes both longitudinal and lateral control into consideration. The benefit of platooning is linked to the distance between two following vehicles, i.e., the inter-vehicle distance. The inter-vehicle distances are down to approximately 10 m, which is smaller compared to human driving. This reduce aerodynamic drag and thus, energy consumption. Even smaller distances do not reduce energy consumption significantly more but may introduce cooling problems, especially for heavy goods vehicles (HGVs) [1]. Although platooning is mainly considered for HGVs, it is not necessarily limited to them.

The aerodynamic drag is responsible for approximately a quarter of the energy consumption for an HGV. This causes approximately one third of vehicle life cycle costs. According to the industry, any technology for long-distance haulage that promises energy savings is already considered to be worth investigating from a level of 0.5% [2].

Research has shown that the energy-saving potential for various vehicle types is significantly above the 0.5% savings threshold [3]. Depending on the position within the platoon, energy savings between 2.8% and 9.7% per vehicle are possible. Even the leading vehicle benefits from platooning and saves between 2.7% and 5.3% consumed energy. Team energy savings, i.e., savings of all vehicles within one platoon, at a distance of approximately 9 m and a velocity of approximately 90 km/h^−1^ reach 3.7–6.4% [4].

Furthermore, platooning augments the road capacity, i.e., the maximum number of vehicles per unit of time that have a reasonable expectation of passing over a particular roadway component under prevailing roadway and traffic conditions [5]. Depending on inter-vehicle distance and the number of platooned vehicles [6], road capacity can be significantly increased due to the closer spacing and smoother driving behavior among vehicles within the platoon. The authors show as an example, in [7], that road capacity is increased by 11.5% when only 12% of the vehicles are in platoons of six vehicles. Higher percentages of platooned vehicles or platoons consisting of more vehicles further increase road capacity.

Platooning is more than longitudinal and lateral automation. However, status and intent must be shared, and agreements need to be found [8]. Platooning can be seen as a three-layered system [9]: the transport layer distributes the goods over the available vehicle capacities and assigns their routes. The platoon layer performs platoon management, i.e., forms, maintain, dissolve, and perform maneuvers. Finally, the vehicle layer performs real-time vehicle control, including longitudinal and lateral control, and is responsible for safe operation.

All layers require communication either between infrastructure and vehicle or between vehicles. Different layers have varying requirements on the quality of service of the communication network. Requirements with respect to availability, reliability, end-to-end delay, and data-freshness go hand in hand with the impact of each layer in the safe operation context. For example, data on the vehicle layer is of small payload as it mainly contains vehicle state information, but it must be sent with high frequency and received on a timely and reliable basis. In contrast, data on the transport layer has a greater payload but needs to be sent less frequently. Package losses here can be absorbed and compensated for.

## 2. Related Work, Contribution, and Outline

In this section, we discuss existing work in the field of vehicular communication within a platoon, point out research gaps, name our main contributions, and describe the outline of our work.

### 2.1. Related Work

The performance of various communication technologies in a vehicular environment has been discussed and evaluated in a wide range of works, as well as with respect to platooning. IEEE 802.11p and mobile networks (LTE/5G), in addition to non-radio frequency-based technologies such as visible light communication (VLC), are especially of interest. One single conclusion on which technology is superior, however, cannot be established [10]—its selection depends on many factors. The authors in [11] see 5G as a promising solution to meet the low-latency and high-reliability requirements of platooning—whether the promise can be kept remains to be seen. A few works combine two communication technologies and perform hybrid networking. The authors in [12] combined IEEE 802.11p and LTE in an exemplary manner and selected the optimal communication technology on a per-packet basis for data dissemination in a vehicular network. Han et al. [13] showed, however, that IEEE 802.11p demonstrates poor performance in highly populated and dense networks. Segata et al. [14] indicated severe impairments related to resource scheduling in LTE once used for V2X communication leading to packet loss. This means that despite selecting the currently optimal technology, it may still not be adequate for the task. Other communication technologies may offer a promising solution: the authors in [15] used VLC as a backup and offloading communication technology to IEEE 802.11p. Similarly, VLC and IEEE 802.11p were combined in [16] using network-selective message forwarding dependent on network load. However, both focus only on exchanging data used for inter-vehicle distance control.

Irrespective of the network type, a higher load on the network endangers the quality of service provided [17,18] with the result that platooned vehicles are forced to increase their inter-vehicle distance [19]. Hybrid communication can improve the quality of service in the event that communication channels are uncorrelated. However, this requires sophisticated management to ensure that the needed data are exchanged between the communication partners in time. This also includes data from the transport and platoon layer that are not directly related to inter-vehicle distance control.

A fact that is often forgotten when focusing on the inner working of a platoon is that the surrounding non-platooned traffic must also be considered. On the one hand, it must not be disturbed, e.g., by blocking lane entries or exits; on the other hand, it poses a risk for the operation of the platoon: cut-in maneuvers, lane switches, overtaking, or accidents need to be considered. Platoons may be able to react to certain scenarios [20,21]. However, in cases where inter-vehicle distances need to be adapted and especially when this affects multiple platooned vehicles, the reaction is simply too slow. Unforeseeable events and events that lie outside the field of the platoon levels require explicit consideration and handling to ensure that their impact is limited [22]. Further to this, the information on non-platooned traffic may be vague since appropriate communication may not be supported. This limits the possibility of constructive reaction. Neglecting network capabilities, from a platoon perspective, and selecting a very small inter-vehicle distance would improve safety as cut-in maneuvers are inherently avoided. In addition, this would increase efficiency. However, the complexity and difficulty of platoon maneuvers would be increased. The dissolution of a platoon would be a long ongoing process until the inter-vehicle distance is reached when human drivers could take over [23]. From a non-platoon point of view, a large inter-vehicle distance would be preferred as it reduces the traffic disturbances caused by the platoon.

Ultimately, platoon efficiency is highly linked to the inter-vehicle distance achieved and a driving behavior without braking maneuvers. Platoons influence and are influenced by surrounding traffic. This interdependency may require inter-vehicle distance adaptations to guarantee safety. Furthermore, platoons rely on a robust and timely network that may not always be present. This, again, may necessitate adapting the distance between the vehicles in platoons.

### 2.2. Contribution

In contrast to existing work [24], we do not only validate the applicability of individual communication technologies for platooning, nor do we only consider hybrid networks for the vehicle layer. The main contributions of our work in this paper are listed as follows:

1. We present *Feudalistic Platooning*, an extensive platooning concept including a vehicle and a platoon layer. It uses hybrid networking targeting both safety- and efficiency-related bottlenecks caused by communication deficiencies and surrounding traffic. Within a platoon, the message flow is steered by diversified role-based communication which unites either two only or alternatively many more communication networks and technologies.
2. We employ IEEE 802.11p and cellular V2X (C-V2X) and discuss how to improve operability, coexistence with surrounding traffic, and modularity, to relieve networks, to compensate network outages, and to increase efficiency. For C-V2X, we do not consider 5G but LTE. However, these are interchangeable.
3. We simulate *Feudalistic Platooning*, analyze the data flow and load using a comprehensive, open source suite for inter-vehicle communication simulation, and discuss its impact.

### 2.3. Outline

To do this, we recap ad hoc and cellular-based communication in Section 3. We assess the merits and shortcomings of the networks with respect to vehicle use. We also discuss the need for hybrid solutions and the special, challenging requirements of platooning. Subsequently, Section 4 proposes the concept of *Feudalistic Platooning* and elaborates the steer of the message flow. In Section 5, we simulate the proposed approach using Omnet++ and Simulation of Urban Mobility (SUMO) and discuss the results, especially those regarding the elaboration of the data load and flow. Based on this, we indicate the key advantages of *Feudalistic Platooning*. Finally, in Section 6, we conclude the work, discuss the limitations, and highlight potential future work.

## 3. Vehicular Communication

Two types of communication technologies are now in use to exchange data between two or more nodes in vehicular environments: these are ad hoc or cellular networks. Before we discuss each network in detail, we briefly introduce the following concepts of unicast, broadcast, and multicast:

- Unicast: data are sent from the transmitter to one receiver using a dedicated link. In the event of multiple nodes receiving the same message, the same number of transmissions and links as receivers are necessary.
- Broadcast: data are sent from the transmitter over a single link that is shared by *all* nodes. Even though only one transmission is necessary, all nodes are able to receive the data.
- Multicast: similar to broadcast, but the link is only shared by *some* nodes. Geocast is a special form of multicast, where nodes are identified by their geographical locations.

### 3.1. Ad Hoc Network

Build on top of the IEEE 802.11 specification, the IEEE 802.11p standard forms the basis for vehicular ad hoc communication within the 5.9 GHz band, and this was already published in 2010. Communication within the ad hoc network is decentralized, meaning that no entity is governing the communication. As it is not bound to any infrastructure, it is available everywhere and therefore cost-effective. The communication range is very limited when a high packet reception rate (PRR) is required, e.g., data exchange on the vehicle layer. Considering small data packets of 100 bytes, a communication distance below 50 m is required to reach a PRR above 90%. Increasing the packet size significantly decreases the PRR [25]. Messages are usually broad- or geocasted. Multiple communication channels are available for this purpose [26]. Of course, for successful communication, receiver(s) and transmitters need to use the same channel, which they should agree before the data exchange.

Transmitting nodes need to follow the CSMA/CA (carrier sense multiple access/collision avoidance) concept. This means that once a node wants to send a message, it listens on the channel for already ongoing communication. If none is detected, the node can start to transmit immediately, otherwise it waits for a random back-off time. This simple “listen before talk” principle prevents some collisions. However, packet collisions may occur due to phenomena such as the *hidden node* problem. Furthermore, the channel utilization is non-optimal as nodes may wait where it would not be necessary, causing an accumulation of data that still need sending. For example, this occurs in *exposed station problem* scenarios.

Reliability is lost once collisions occur, and timeliness is harder to achieve once the network is non-optimally utilized. This fact worsens once the load on the network increases. The load on a network is increased by increasing the number of transmitting nodes, the payload size, and the number of packets. The latter may grow exponentially in the case of packet forwarding, e.g., to reach nodes outside of direct communication range, or in case retransmissions are necessary. An already battered network harms itself by requiring retransmissions.

### 3.2. Cellular Network

In contrast to the decentralized approach in ad hoc networks, each cell within a cellular network is governed by a base station. This means that communication is only possible once infrastructure is available—at least in the classical sense, but more on that shortly. Limited cell size has the consequence that moving nodes may move outside the covered area, i.e., the cell. The new area may be part of a new cell or may not be covered at all. In the first case, cellular handover needs to be performed, which usually leads to delays of approximately 50 ms [27], where the node is not accessible. In the second case, communication is no longer possible. Connection can be established again upon re-entry into a cell. Communication within a cellular network needs to obey strict rules. Channels are divided into small resource blocks that are assigned to nodes that ask for communication permission. No node is allowed to send without permission. This increases the reliability enormously as packet collisions are mostly avoided and further, allows good network utilization [18]. Still, network coverage may not be always given.

Typically, communication takes place over long distances—the radius of a macro cell can be up to 25 km [28], although in vehicular environments, most of the time, communication partners are located at short distances. Additionally, communication always runs via infrastructure nodes resulting in higher latency compared to ad hoc networks unable to meet the strict delay requirements for safety applications. A transmitting node sends its data as unicast to the base station. High vehicle density may overload this uplink channel. Once received by the base station, it either unicasts, multicasts (geocasts), or broadcasts the message for receiving nodes. Due to the size of cells, broadcasting may result in the reception of many irrelevant messages that need to be further processed by each vehicle. Multicasting comes along with high control signaling overhead and high latency. Once unicasting, e.g., to relevant vehicles only, the downlink channel becomes a bottleneck that already has a few vehicles [29].

To overcome latency issues and avoid overloading the up- and downlink channels, cellular V2X (C-V2X) was added to the Long-Term Evolution (LTE) standard. C-V2X is specified in Release 14 in 2017 and enables two modes for direct communication between nodes in- and outside of network coverage: Mode-3 and Mode-4. Mode-3 targets direct communication under coverage and supports radio resource management. The latter is not offered by Mode-4, but this mode does not require network coverage [19].

### 3.3. Hybrid Networking

Currently, both ad hoc and cellular networks are struggling to fulfill vehicular communication requirements that are highly application dependent. Varying the quality of service requirements can hardly be considered for communication, since only one single network is used. The mismatch between requirements and available communication technologies increases the desire for multiple network usage [29]. However, once multiple networks are combined, mutual interference must be excluded so that their coexistence can be first made possible [30]. Furthermore, the adding of simple redundancy may not be sufficient for this task. Hybrid solutions for data dissemination will need to be sophisticated in order for them to be capable of relieving the individual network and ensuring sufficient quality of service [12]. Clustering allows the network load to be reduced even further [31]; however, a trade-off in cluster design needs to be found: large clusters lead to a high network load within a cluster and low network usage between clusters. In [32], the current context of the vehicle is observed and the optimal communication technology is selected on a per-packet basis. In addition to this, a reputation system estimates trust scores for the hybrid system.

### 3.4. Platooning

Performing platooning in both ad hoc and cellular networks is a challenging task. A great many small, periodic data packets need to be sent in a reliable and timely manner to satisfy the vehicle layer needs. Very intensive periodic messaging is required to keep the data fresh, but this challenges the capacity of the ad hoc network and is even more challenging for the cellular network’s capacity [33]. Multicasts and broadcasts can be a further reinforcement here. Multi- or broadcasts are convenient because the same data packets need to be received by many different vehicles. Unicasts significantly increase the transmission effort.

In addition to periodic messages, event-triggered messages for platoon management are also involved [20]: Platooned vehicles agree on some structures and react on the input received from selected vehicles. For example, vehicles agree on a leader and adjust their speed according to a predecessor(s)/leader control policy. Neighboring nodes may increase the load even further by transmitting data in the same network and channel. Long platoons may need to consider packet forwarding as not all nodes may be in direct communication range. Typically, inter-vehicle distances are short, meaning that delays need to be kept small.

Vehicles send cooperative awareness messages (CAMs) [34] and decentralized environmental notification messages (DENMs) [35] to notify their presence and detected events or even use collective perception messages (CPMs) [36] to share information about objects in the surroundings. All vehicles in the communication range receive messages and may process them. Platooned vehicles may react to messages from vehicles of interest, which depend on the selected control policy [37]. For example, a vehicle may adapt its velocity according to the position, velocity and acceleration included in a message from its predecessor. In contrast, the same message from its follower may be ignored. In the event of a packet loss, e.g., due to collisions, crucial information for the stabilization of the platoon is lost, meaning that it may no longer be possible to safely maintain inter-vehicle distances.

To summarize, a network enabling efficient platooning needs to provide high reliability, high availability, short end-to-end delays and data-freshness.

### 3.5. Nomenclature

The following nomenclature is used: *ad hoc communication* describes the communication based on IEEE 802.11p. *LTE communication* denotes communication using the cellular infrastructure only, where the Long-Term Evolution (LTE) standard is applied. *C-V2X communication* refers to direct vehicle-to-vehicle communication either in Mode-3 or Mode-4 in LTE.

## 4. Feudalistic Platooning

*Feudalistic Platooning* targets both the vehicle and the platoon layer, meaning that it considers data exchange for both real-time vehicle control and platoon management. To do this, *Feudalistic Platooning* combines ad hoc, LTE, and C-V2X networks. By the redundant transmission of highly critical data, i.e., data needed for vehicle control, and the diversified transmission of the other remaining data, it ensures efficient platooning. Furthermore, this redefines the understanding of a platoon: a feudalistic platoon is no longer a simple convoy of vehicles, following each other according to a control policy. It is a system of small platoons that are united within a larger platoon. Participating vehicles adapt their inter-vehicle distance and send messages according to roles. On the one hand, this allows the facilitated integration of the platoon in the surrounding traffic and limits their interdependency. On the other hand, it determines the message flow, eliminating the unnecessary packet forwarding and retransmission.

### 4.1. Background

The feudalistic system during the medieval period was characterized by strict hierarchies and mutual obligations, and the strict roles and responsibilities associated therewith. The number of people in each hierarchy layer is increased from top to bottom. We can thus visualize the system as a pyramid: its base is composed of peasants and serfs (I) followed by craftsmen and merchants (II). The knighthood comprising knights and vassals (III) finds its place above these. They are under obligation to lords and nobles (IV) who are in turn under the monarchy, usually represented by a single emperor or king (V). Above all monarchies stands the church (VI), represented by the pope.

Hierarchies and roles are advantageous when all the entities of a system are not performing the same tasks. In communication networks, roles are often assigned according to the capabilities of single nodes, e.g., only a few nodes may have enough computational power to perform certain tasks. Furthermore, within a hierarchy layer, tasks may be split to reduce complexity.

This feudalistic view can also be applied on already platooned vehicles to amplify data obligations, to control the message flow, to decrease the network load, and finally, to increase reliability. We denote this as *Feudalistic Platooning* and it affects both the vehicle and the platoon level.

### 4.2. Concept

*Feudalistic Platooning* does not substitute a general platooning concept for the platoon level and control level—extends it instead. Furthermore, it is able to combine two or more networks. In the following, we describe *Feudalistic Platooning* on the back of ad hoc (IEEE 802.11p) and cellular networks (LTE and C-V2X). However, networks are interchangeable. Moreover, cellular coverage is not a prerequisite for an operational *Feudalistic Platooning* system: occasional and partial availability is sufficient.

**Figure 1:**
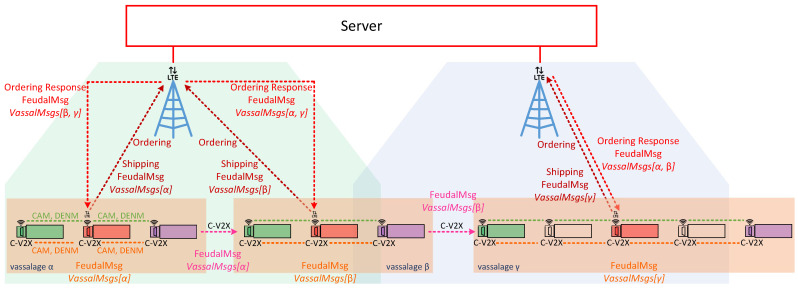
*Feudalistic Platooning*: Vehicle group as vassalages of variable size that act according to specific roles: knight (green); lord (red); merchant (purple); and peasant (no color).

LTE is used to access a central server (VI). This means that the central server may be accessed from every cell (V) (see Figure 1). It collects and stores platoon information. Furthermore, it is the source of its distribution. A more detailed description of the central server can be found in Section 4.5.

An already existing platoon may activate and also deactivate *Feudalistic Platooning* at any time. To perform *Feudalistic Platooning*, the platoon is divided into smaller convoys of vehicles, so-called vassalages. Each of those vassalages consists of at least three, and up to many platooned vehicles. Within a vassalage, each vehicle acts according to specified roles that depend on their position within the vassalage. Independently of their role, all vehicles emit vehicle status information in terms of CAMs and notify events using DENMs. They send both types of information redundantly using the ad hoc and C-V2X networks. Once performing *Feudalistic Platooning*, the ad hoc network is used exclusively for vehicle status and event information. Received packets of this type are not forwarded. We distinguish between four different roles, where roles IV, III, and II need to be occupied:

- Lord (IV): the mid vehicle of the vassalage responsible for shipping data, i.e., it collects vehicle status and event information from all vehicles within its vassalage which it regularly reports to a central server. Furthermore, it performs ordering, i.e., it periodically requests information from other vassalages at the central server. Cellular coverage is required to do this. Received data may be shared among all vehicles of the vassalage using C-V2X that is available both in and outside of cellular coverage. The selection of the mid vehicle ensures the shortest communication paths. One of the two mid vehicles may be selected in the event of an even number of vehicles in the vassalage.
- Knight (III): the first vehicle of the vassalage receives data over C-V2X from the merchant of the preceding vassalage when there is one in this position. It may distribute the received data among all vehicles within the vassalage, again using C-V2X. The selection of the first vehicle ensures the closest communication distance to preceding vassalage.
- Merchant (II): the last vehicle of the vassalage which collects status and event information from all vehicles within its vassalage which it sends to its follower, i.e., the knight of following vassalage—if there is one. C-V2X is used to transmit the data. Selecting the last vehicle ensures the closest communication distance to the following vassalage.
- Peasant (I): any remaining vehicle within the vassalage which does not have to handle additional communication tasks.

A further two additional roles are introduced during the setup phase:

- Claimant: the vehicle part of a platoon and ready to perform *Feudalistic Platooning*.
- Outlaw: the vehicle part of a platoon not ready/capable of performing *Feudalistic Platooning*.

Vehicles within a vassalage perform dynamic platooning [20]: due to data redundancy, they may be able to drive at smaller inter-vehicle distances compared to a normal platoon within the same environment. Exclusive usage of the ad hoc network for vehicle status and event information ensures the best reliability possible. The redundant link may increase the network’s quality of service so that it is able to support smaller inter-vehicle distance. If necessary, inter-vehicle distance may be adapted by dynamic platooning. In contrast, a larger gap is maintained between vassalages compared to inter-vehicle distances of normal platooning. On the one hand, this accounts for the missing data redundancy between vassalages. On the other hand, it creates room for the surrounding traffic. Again, this gap may be dynamically adapted by performing dynamic platooning [20].

### 4.3. Messaging

All vehicles within a platoon (and a vassalage) send CAMs according to [34]. Depending on the driving context, the time interval may vary between 0.1 s and 1.0 s. In case special events occur, DEN messages may be sent according to [35]. If vehicles further want to enable cooperative sensing, CPMs may be exchanged according to [36]. For platoon management (join, leave, split, merge), messages following the proposal in [20] are exchanged. Once a platoon is formed, platoon management mainly consists of periodic messages that keep the platoon updated. While performing *Feudalistic Platooning*, no further periodic messages are needed in terms of management. Instead, *Feudalistic Platooning* introduces *FeudalMsg*, the core of the communication within a vassalage.

**Figure 2:**
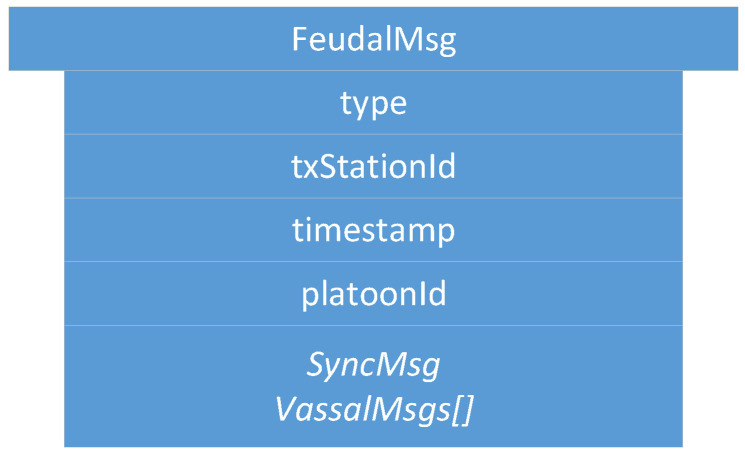
*FeudalMsg*: used for all ongoing communication during *Feudalistic Platooning*. The selection between *SyncMsg* and *VassalMsg* is context dependent and indicated by the type.

*FeudalMsg*:

The *FeudalMsg* (see Figure 2) is a modular message that currently encapsulates one of two different messages: *SyncMsg* and *VassalMsg*. From the latter, multiple messages may be present within one *FeudalMsg*. Which of the two messages is included within *FeudalMsg* is indicated by *type*. Furthermore, the message contains the ID of the transmitter (*txStationId*), a time stamp (*timestamp*), and the platoon ID (*platoonId*). The latter helps to resolve ambiguities in the event that more than one platoon is within communication distance.

**Figure 3:**
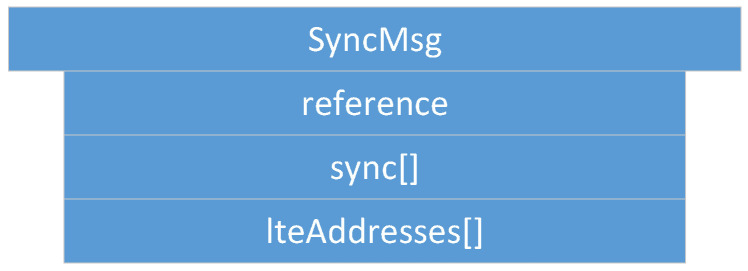
*SyncMessage*: all vehicles in a platoon need to synchronize to the same request (reference) prior to perform *Feudalistic Platooning*, i.e., agreeing on *Feudalistic Platooning* (sync[]) and sharing their LTE address (lteAddress[]).

*SyncMsg*:

It is part of *FeudalMsg* and included once *FeudalMsg* is of type *::Sync*. *SyncMsg* is used during the synchronization process (see Section 4.4) to successfully devolve from an ordinary platoon to a feudal platoon. As illustrated in Figure 3, *SyncMsg* consists of a reference number (*reference*), a Boolean sync array (*sync*), and an array for cellular LTE addresses (*lteAddresses*). Both arrays have the same size as the number of vehicles that are part of the platoon.

**Figure 4:**
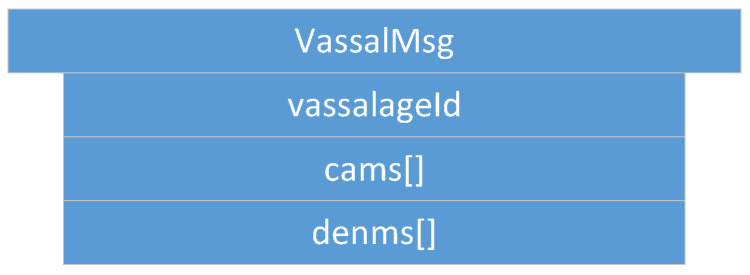
*VassalMsg*: containing vehicle status (cams[]) and event (denms[]) data from vehicles within a certain vassalage indicated by *vassalageId*.

*VassalMsg*:

It is part of *FeudalMsg* and included once *FeudalMsg* is of type *::Vassal*. Including one *VassalMsg* is then compulsory. *VassalMsg* is exchanged to share information about a vassalage. Many *VassalMsgs* may be included, e.g., once information from other vassalages of the platoon is shared. As shown in Figure 4, *VassalMsg* consists of the vassal ID of the originating vassalage (*vassalageId*) and arrays for CAMs and DENMs containing all the vehicle status and event information from the vassalage members (*cams*, *denms*).

### 4.4. Synchronization

**Figure 5:**
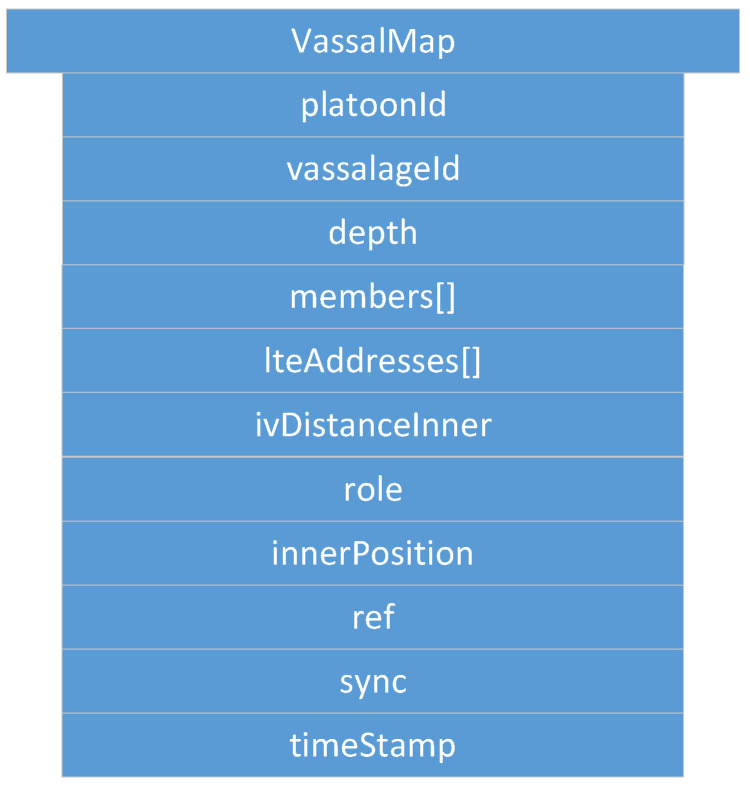
*VassalMap*: locally stored on each vehicle, containing information needed to perform *Feudalistic Platooning*.

Data needed to perform *Feudalistic Platooning* are locally stored by each vehicle in a *FeudalMap* (see Figure 5). In order to not depend on cellular coverage or any addressing within the cellular network, we use the ad hoc network for synchronization. The synchronization process initiates and updates fields within the mentioned map. It finishes, once all vehicles have stored the required data. The vassalage is uniquely identified by its ID *vassalageId* and the corresponding ID of the platoon *platoonId* it belongs to. The *VassalageMap* also contains the number of vehicles within the vassalage (*depth*) and their IDs and addresses (*members*, *lteAddresses*).

*Note: members* and *lteAddresses* include the merchant of the preceding vassalage if there is one as well as the knight of the following vassalage, again if there is one. Their IDs and addresses need to be known once communication between vassalages based on C-V2X is considered. Neither of them is compulsory, however, for an operating feudalistic platoon.

Furthermore, each vehicle selects an individual inter-vehicle distance (*ivDistanceInner*) which depends on its position within the vassalage (*innerPosition*), its role (*role*), and external circumstances considered by dynamic platooning [20]. Every time data are modified, the time stamp (*timeStamp*) is updated.

As with platooning in general, *Feudalistic Platooning* should also be decentralized. This means that required data differ from vehicle to vehicle. The platoon or a part of the platoon determines either a fixed or a variable vassalage size. Future lords, i.e., the mid vehicle of future vassalages, initiate the formation procedure by sending a *FeudalMsg* of type *::Init* (see Figure 2). The message contains IDs and LTE addresses of future vassalage members, i.e., the size of the vassalage plus two: (i) the merchant from the preceding vassalage and (ii) the knight from the following vassalage, as mentioned previously. In case no preceding or following vassalage exists, the field remains empty. The IDs are known on initiation, since the vehicles need to be platooned. By contrast, the LTE addresses of other members do not need to be known. The initiator sends its LTE address in the broadcasted *::Init* message and keeps address fields of other members 0. By means of the LTE address, a vehicle is uniquely identified within the cellular network. Receivers of the message check if the message is of relevance, i.e., their ID is contained in the member field. If this is the case and their address was not already present in the receiving packet, they add their own address and rebroadcast the message. This ensures that messages are only present once new information is available and unnecessary flooding of the network is avoided. Furthermore, receiving vehicles check whether they are core or peripheral members:

- Core members are lord, knight, merchant and all the peasants of the dedicated vassalage;
- Peripheral members are merchant and knight of preceding, respectively, the following vassalages.

Addresses of peripheral members need to be known to exchange messages between vassalages using the C-V2X network link.

In addition to adding its address and rebroadcasting the packet once new information is available, core members determine their own role within the newly founded vassalage according to their track position. Furthermore, they checked whether all vehicles that wish to be part of a dedicated vassalage are ready, i.e., all LTE addresses are present. If so, they set role-specific parameters, e.g., selecting new inter-vehicle distance and start their role timers. The latter determines the frequency with which communication takes place (see Section 4.6). In the event that the core members are not yet ready, a synchronization timer ensures the rebroadcasting of the *SyncMsg* after a specific time lapse. This guarantees that the synchronization process does not fail due to lost or corrupted packets.

*Note:* the concept of core/peripheral membership allows that knights and merchants may be part of two vassalages. Nevertheless, only one core/peripheral membership is present at any point in time.

The presented approach is decentralized, i.e., only addresses from core and peripheral members of the vassalage are needed for synchronization. Furthermore, a central, platoon-wide exchange of data could be used. The following points explain why a decentralized approach may be favored:

- Synchronization time: as at least every vehicle needs to send one packet, having fewer vehicles that need to exchange data accelerates the synchronization process.
- Network load: the required data are from vehicles that are geographically very closely grouped. This ensures short communication distances, avoiding packet forwarding to reach all vehicles of the platoon: most of the time, a single packet transmission by a vehicle may be sufficient.
  Furthermore, in busy networks, the transmission power could be reduced to such an extent that only very few vehicles are actually able to receive the messages. This may lead to packet forwarding but with small vassalage sizes, synchronization is still possible at a decent speed.
- Robustness: less packet forwarding and less vehicles that need to synchronize increase the reliability of successful, collision free packet transmission and does not stress the network unnecessarily. This increases the robustness in comparison to that of centralized synchronization.
- Frequency reuse: as communication is geographically bonded to a small area of the platoon, the same frequency (channel) may be reused in another part of the platoon.
- Operability: outside the vassalage, the platoon is still open for platoon maneuvers, e.g., to join, leave, merge, or split.

### 4.5. Central Server

**Figure 6:**
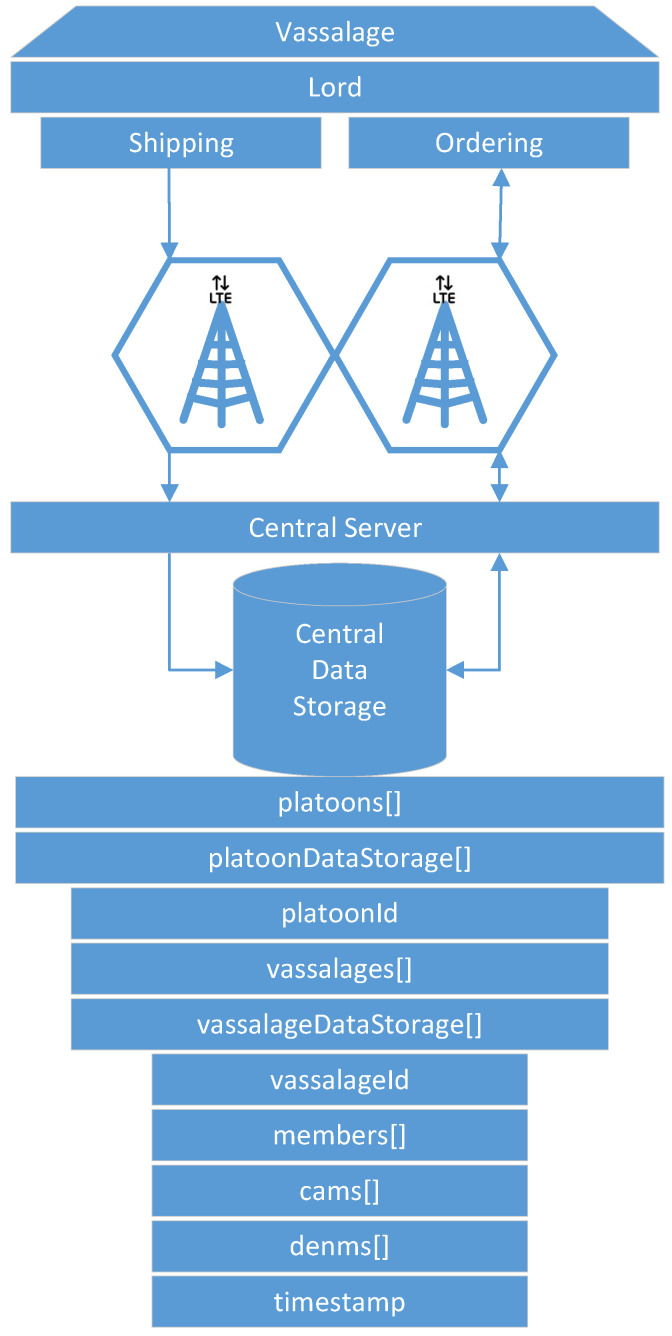
Lord shipping/ordering vassalage data to/at central data storage. Data are stored per platoon and per vassalage.

The central server consists of a central data storage that contains information about platoons and their vassalages. Communication with the server is performed using LTE only. Thus, it is only available under coverage. The data storage may be located far off the roads. From the platoon perspective, lords are the only vehicles that interact with the central server. Lords ship data from their own vassalage to the central server. There, shipped data are stored within a data storage per platoon and per vassalage (see Figure 6). Furthermore, lords may order data from vassalages within their platoon or from neighboring platoons.

*Shipping*:

Lords collect vehicle data, i.e., CAMs and DENMs, from their own vassalage and send it in terms of a *FeudalMsg* containing a single *VassalMsg* (see Section 4.3). Shipped data are periodically refreshed in larger time intervals, compared to CAM dissemination, e.g., every two seconds.

However, shipped data are not critical for the operation of the platoon, as the transmission of highly critical data from surrounding vehicles is ensured using ad hoc and C-V2X networks (see Section 4.2). Of course, the control policy needs to be selected appropriately: no data from vassalages other than the own and preceding vassalage are required. This ensures two decisive things: first, platoon operation is still possible without cellular coverage. Although cellular networks increase the performance of the platoon management, e.g., by allowing more advanced control policies that may include an arbitrary variety of preceding vehicles, they are not necessary for safe platoon operation. Second, the quality of service of ad hoc networks highly depends on data load. By transmitting non-crucial data over a different network, ad hoc networks are relieved and the overall platoon performance is increased.

*Ordering*:

Lords may periodically order data from the central server. Data may be of other vassalages within their platoon or may be of neighboring platoons and their vassalages. The order is a *FeudalMsg*, where the platoon of interest is indicated by *platoonId*. Furthermore, one or more vassalages may be indicated by one or more *VassalMsgs*. There, *vassalageId* indicates the vassalage of interest. Of course, *cams[]* and *denms[]* remain empty. The server responds with the corresponding *FeudalMsg* containing one or more *VassalMsgs*. If the order includes no *VassalMsgs*, the server responds with the corresponding *FeudalMsg* containing *VassalMsgs* from all vassalages of the platoon, excluding the vassalage of the ordering lord.

Responses received from the server add redundancy to the C-V2X link between two vassalages. Furthermore, very long platoons may benefit from the information received from vassalages located upstream that is used to anticipate control actions. Furthermore, control policies using preceding vehicles that are not part of the own vassalage, e.g., that include the leader, may rely on data from the central data storage. Above that, in the future, data from neighboring platoons may be used to reconcile actions between platoons, e.g., finding common destinations to prepare a reorganization of single vehicles and/or single vassalages within a platoon on freeway junctions.

Alternatively, the central server may periodically update lords about other vassalages. However, our described approach has the following advantages: first, lords may adapt their request interval according to the quality of different networks. Second, lords decide which data are relevant and can explicitly ask for it. Third, lords may ask data from certain vassalages at higher frequencies compared to data from other vassalages. For example, the data used for inter-vehicle distance control may require higher frequency.

### 4.6. Timers

Dependent on the message, within a feudalistic platoon, message transmission is either time or event triggered. Event-triggered messages include DENMs and messages sent during synchronization (see Section 4.4). CAMs are sent periodically according to [34]. However, their transmission frequency may vary. Furthermore, *FeudalMsgs* are sent periodically. To trigger their message generation, the following timers are used. They may be selected statically immediately after synchronization or may be updated once needed.

*Merchant Interval*:

The merchant interval determines the time between two consecutive messages sent by the merchant to the knight of the following vassalage if they exist. A shorter interval increases the load on C-V2X and favors data freshness within the following vassalage.

*Ordering Interval*:

The ordering interval determines the time between two consecutive orders sent by the lord to the central server. A shorter interval increases the load on LTE. Note that this does not only affect the load of ordering itself, but also the load of the corresponding responses from the central server. Consequently, it favors data-freshness within the vassalage.

*Shipping Interval*:

The shipping interval determines the time between two consecutive messages sent by the lord containing the vassalage’s data. A shorter interval increases the load on LTE. It favors data-freshness on the central server and further in each vassalage that requests these data.

### 4.7. Vassalage Size

The size of the vassalage impacts platoon efficiency and places various requirements on the network and traffic with respect to safety. Fewer vehicles within the vassalage allow a better integration of the platoon in situations of high surrounding traffic volume, e.g., in urban areas. Demands on the quality of service of the ad hoc network are reduced, as a smaller amount of transmitting vehicles is present compared to larger vassalage. In case a low packet reception rate is observed, smaller vassalages should be chosen in advance. This relieves the ad hoc network and pushes the load more onto the LTE network, which is usually available in good quality near urban areas. However, this comes at the price of efficiency losses across to the whole platoon, such as increased aerodynamic drag. In contrast, a larger vassalage could be selected in situations with low surrounding traffic volumes, e.g., in rural areas. Moreover, the size of vassalages within a platoon may be dynamic to react to these changing circumstances.

Furthermore, the vassalage size may differ from vassalage to vassalage, e.g., in favor of common destinations. This facilitates the extraction and integration of single vassalages from one platoon into another, for example, at freeway junctions.

## 5. Results

To validate and analyze the data flow within *Feudalistic Platooning*, we implemented the presented concept and simulated a platoon consisting of twelve vehicles. As a simulation environment, we used OMNeT++ Discrete Event Simulator coupled over the TraCI interface with Simulation of Urban Mobility (SUMO). Within OMNeT++, Artery [38] enables V2X communication based on IEEE 802.11p, and SimuLTE [39] enables V2X communication based on LTE and C-V2X. SUMO, a microscopic traffic simulation, accounts for vehicle and traffic dynamics. Together, they offer a comprehensive, open source suite for inter-vehicle communication simulation, targeting both ad hoc and cellular networks. We evaluate simulation results, discuss key advantages, and explain how existing platooning concepts could benefit from *Feudalistic Platooning*.

### 5.1. Simulation Scenario

Vehicles travel on a motorway supported by one single cell of a cellular network. All vehicles continuously transmit CAMs. Table 1 summarizes chosen the simulation parameters: a vehicle drives with a *constant speed* of 30 m s^−1^ and no situations worthy of announcement occur. Hence, no DENMs are transmitted. The platoon is formed decentralized according to [20]. Once platooned, vehicles perform *Feudalistic Platooning*. To do so, the twelve vehicles group into four equally sized vassalages, each consisting of one lord, one knight, and one merchant. During the simulation, the vassalage size is not adjusted. As *merchant interval*, we select 1 s, i.e., the greatest possible interval in which a vehicle is transmitting CAMs, with the effect that vehicle state data from the preceding vassalage could be considered for vehicle control if necessary. Data from vassalages further upstream are less critical in terms of time: on the one hand, data may not be relevant for the selected control policy. On the other hand, the spatial distance is increased as at least one vassalage is in between. Thus, the *shipping interval* and *ordering interval* should be selected according to control needs and may be dynamically and individually adapted based on proximity to the relevant vehicle, e.g., the leading vehicle. In this scenario, a static interval of 2 s is used for both shipping and ordering. *Intra-Vassalage Vehicle Distance* is small enough that a vassalage cannot be divisible by the surrounding traffic: Yang et al. [40] showed that for a successful lane change on a freeway, the lead and lag gap combined need to be at least 0.27 s. Furthermore, the length of the cutting vehicle has to be added. Neglecting the latter and at our speed, this would result in a minimum gap of 8.1 m. The distance between two vehicles of different vassalages (*inter-vassalage vehicle distance*) must be kept sufficiently large to allow surrounding traffic to use the gap when necessary, e.g., to reach the breakdown lane. The median for accepted gap lengths is 2.26 s [40], corresponding to 67.8 m. Thus, we select 70 m as the gap between two vassalages.

Although in the presented simulation scenario, the *intra-* and *inter-vassalage vehicle distance* are statically selected, distance between vehicles may be dynamically adapted: dynamic platooning within and between vassalages accounts for environmental changes [20] and may increase or decrease the gap once needed or possible.

**Figure 7:**
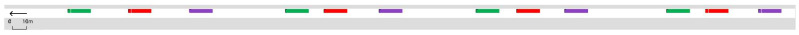
Feudalistic platoon consisting of 4 vassalages visualized in Simulation of Urban Mobility (SUMO). Each vassalage consists of knight (green); lord (red); and merchant (purple).

Figure 7 shows the platoon after a successful synchronization process: the vehicle colors indicate their roles. The depicted scenario shows inter-vehicle distance adaptations caused by *Feudalistic Platooning*: while gaps between vassalages start to increase, gaps between vehicles of the same vassalage start to decrease.

### 5.2. Evaluation

**Figure 8:**
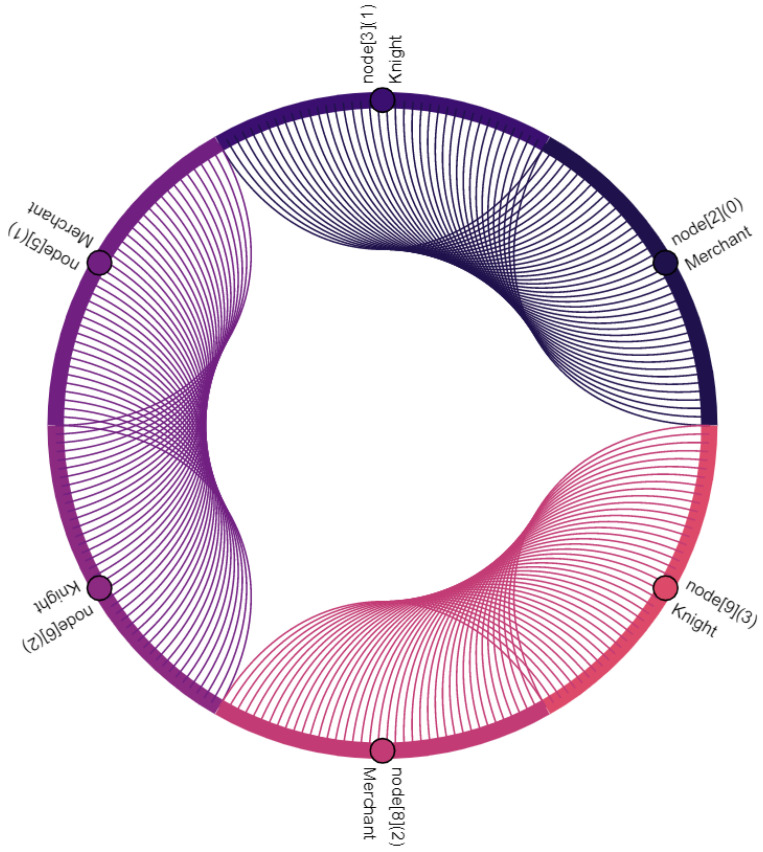
Merchants of each vassalage send their collected vassalage data to the knight of the following vassalage.

**Figure 9:**
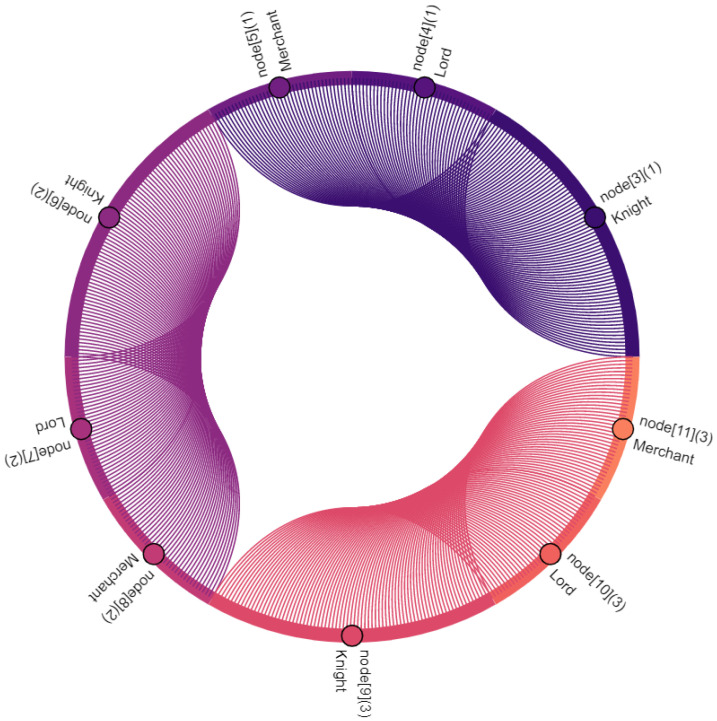
Knights of each vassalage distribute the data from the preceding merchant (see Figure 8) within their own vassalage.

**Figure 10:**
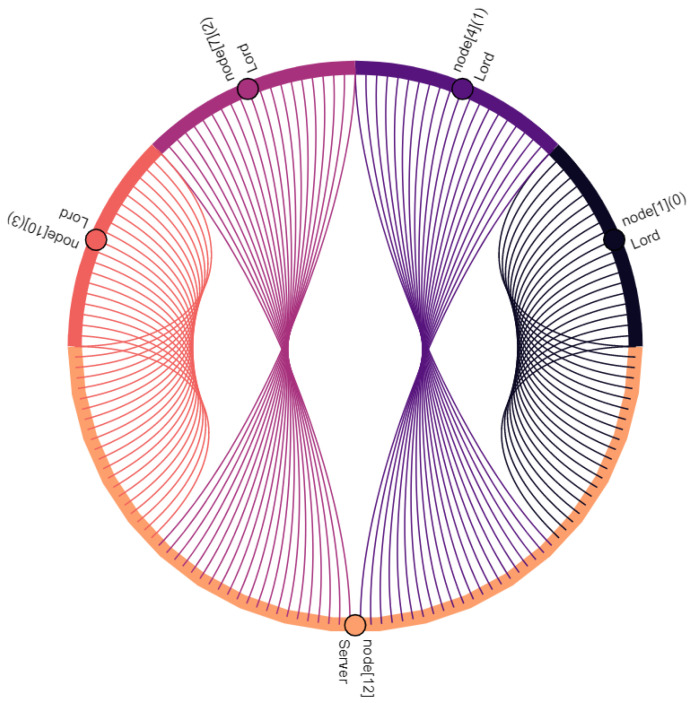
Lords ship vassalage data to central server.

**Figure 11:**
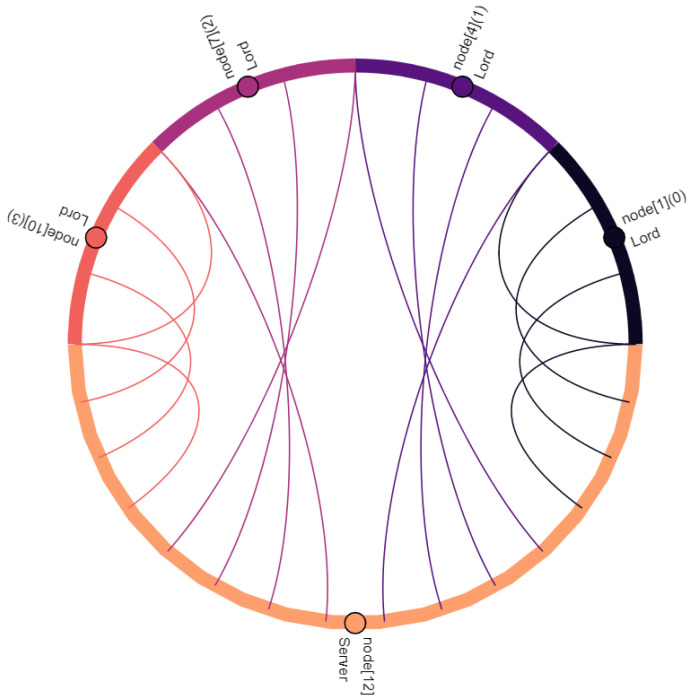
The vassalage lords request data from another vassalage of their platoon at the central server.

**Figure 12:**
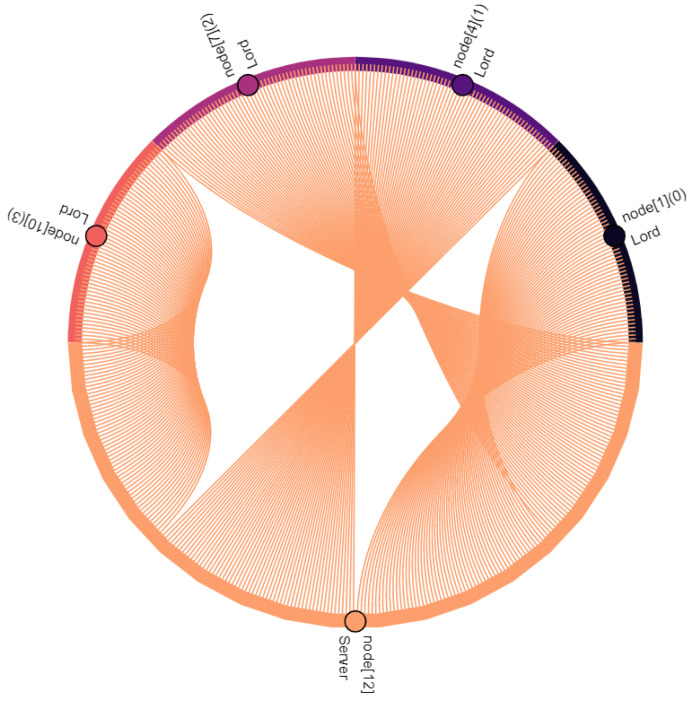
The server replies to the requests of the lords shown in Figure 11.

**Figure 13:**
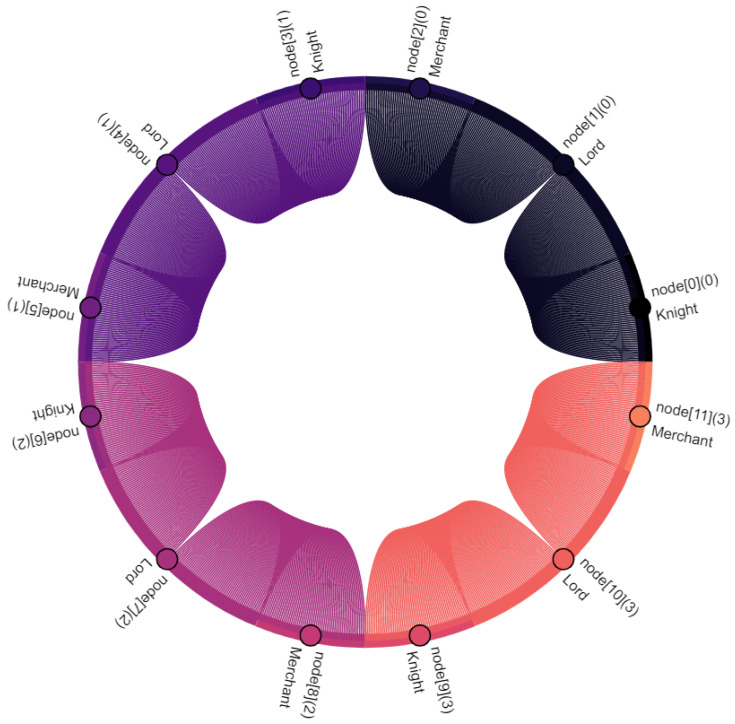
Lords distribute the requested data received by the server (see Figure 12) among all members of the vassalage.

**Figure 14:**
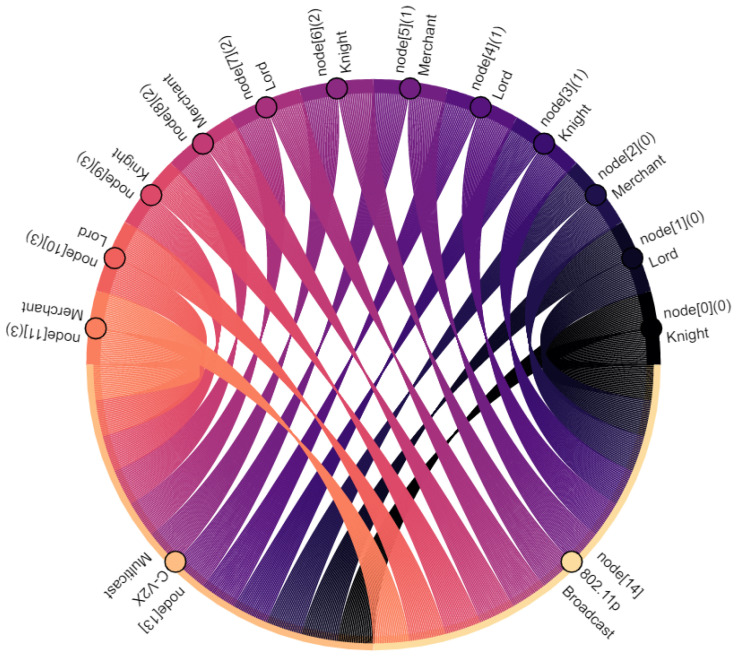
All vehicles broadcast their CAM messages using ad hoc network. The same message is sent redundantly to all vehicles of the platoon (multicast).

**Figure 15:**
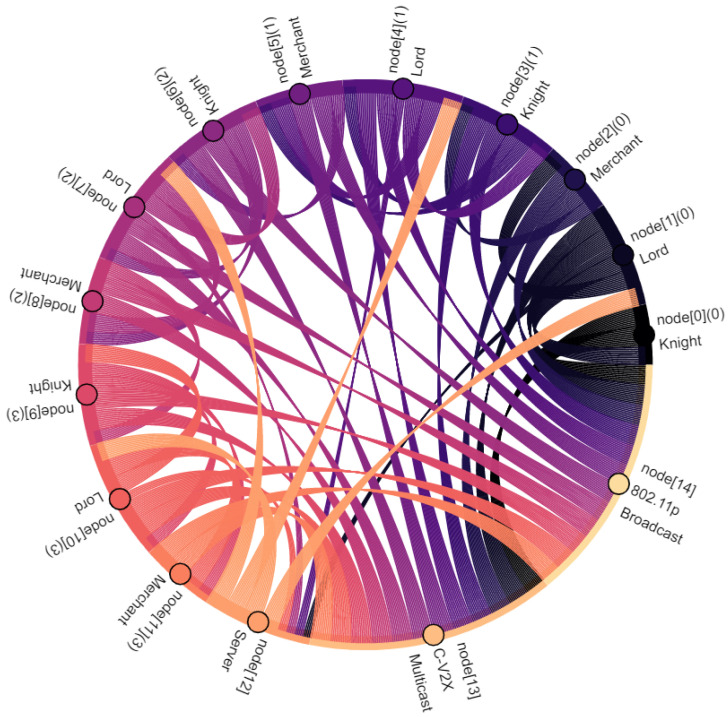
The communication data flow within a platoon performing *Feudalistic Platooning*.

Communication participants, i.e., vehicles and the central server, are denoted as nodes. Vehicles are nodes with indexes 0–11, where the index within square brackets indicates their position within the platoon: node[0] is the leading vehicle, node[11] is the last vehicle of the platoon. Node[12] is the central server. Furthermore, virtual nodes are introduced for broadcast and multicast traffic: node[13] points out multicast traffic in C-V2X. Similarly, node[14] indicates broadcasted traffic in ad hoc network. The affiliation of a vehicle to the vassalage is indicated by the number in round brackets, where vassalages with lower numbers are located further forward in the direction of travel: (0) is the first vassalage and (4) is the last vassalage of the platoon. Figure 8, Figure 9, Figure 10, Figure 11, Figure 12, Figure 13, Figure 14 and Figure 15 show the data flow between all nodes of the network. The color of each node is fixed. Links are weighted based on the amount of payload exchanged between two connected nodes: a single line represents 1 kB.

Figure 8 shows the communication between two vassalages: merchants of vassalages send their vassalage data to knights of the following vassalage. C-V2X is used to do this. Note that communication is one way and only between two neighboring vassalages. Once these data are received by knights, they are distributed within their own vassalage, including all vassalage members (see Figure 9). In this case, the knights forward the data to lords and merchants of their own vassalage—to two nodes in total. Therefore, in comparison with Figure 8, twice the data volume is sent.

Figure 10 shows the shipping of vassalage data to the central server, as discussed in Section 4.5. The data of the vassalage are transmitted by its lord. Lords may also request data from other vassalages. To do so, they send a request to the server indicating which data they are interested in. As illustrated in Figure 11, these requests contain only small data volumes. Requesting data requires significantly less data to be transmitted between nodes compared to shipping, that is sent at the same frequency (see Figure 10 and Table 1).

The server replies to the requests illustrated in Figure 11 with data stored in the central data storage (see Figure 6) from the requested vassalages. As illustrated in Figure 12, these replies contain a relatively large data volume and go directly back to the requesters—the lords. The data size is naturally dependent on the number of vassalages the requesting lord is interested in.

The received data are distributed by the lords among members of their vassalages. Again, in this case, this includes two other vehicles, namely the knight and merchant (see Figure 13).

In addition to *FeudalMsgs*, CA messages are also transmitted. As the velocity remains constant, the generation rate of CAMs is constant [34]. Driving with a velocity of 30 m s^−1^ results in a transmission interval of 0.2 s, which is a characteristic value for free or synchronized traffic on highways [41]. Figure 14 shows the transmission of these messages: vehicles send CAMs redundantly using both IEEE 802.11p and C-V2X. When IEEE 802.11p is used, CAMs are broadcasted. For illustration, these messages are received by the virtual broadcast node[14]. In contrast to that, once C-V2X is used, CAMs are sent as multicast and received by the virtual multicast node[13]. Of course, the payload is the same in both networks.

Finally, Figure 15 and Table 2 sum up all communication within the feudal platoon. Shares are rounded to two decimal places, which may result in minor discrepancies. During 300 s of simulation, *Feudalistic Platooning* was responsible for 2684 kB payload data. The share per vehicle varies between 4.77% (128 kB) and 9.95% (267 kB). CAM transmissions are responsible for 57.22% (1536 kB) of the overall amount of data. This amount of data would also be generated by an ordinary platoon with CAM transmission on two networks. Only 42.78% (1148 kB) are *FeudalMsgs*, used to send or request data to or at the central server, send data to the following vassalage, send data from the central server to the vassalages, and distributing received data within the vassalage. Driving at higher velocity reduces the transmission interval and increases the CAM share even further. For example, if we use the fastest possible transmission of CAMs (0.1 s), then CAM transmissions are responsible for 72.00% of the overall data volume. This shows the limited impact on the data load caused by *Feudalistic Platooning*.

Different shares per vehicles are caused by different roles: Lords transmit the highest data volumes, followed by knights and merchants. The existence of preceding and follow-on vassalages is decisive for the knights and the merchants. Should this not be given, the data volume per role may vary: knights that lack a preceding vassalage do not receive its data and thus, do not distribute data among members of their own vassalage. Similarly, merchants that lack a follow-on vassalage do not need to send data from their own vassalage. Both would correspond to the behavior of a peasant.

Further to this, we can say that intra-vassalage communication results in 5.8 times more data than inter-vassalage communication. This clearly shows the emphasis of *Feudalistic Platooning*.

Furthermore, we observe that only 12.37% (332 kB) of the overall data quantity require cellular infrastructure. As described in Section 4.5, these data are not crucial for the safe operation of the platoon in case of the appropriate selection of an inter-vehicle distance controller. Considering the cellular coverage, especially in mountainous regions and tunnels, it is definitely an important property.

Note that we do not consider data transmitted by the underlying platoon management presented in [20]. Small deviations may occur due to the selected start and end of the simulation which may include/exclude single messages.

### 5.3. Validation and Impact

The successful reception of packets is less likely in a normal platoon than in a feudalistic platoon. The reason for this is first that it may not be possible to reach all vehicles of a platoon of a reasonable length with a single broadcast. As mentioned in Section 3, a distance below 50 m is required to reach a PRR above 90%. When taking into consideration an inter-vehicle distance of 10 m and a vehicle length of 16.5 m, which is typical for HGVs, a platoon consisting of three vehicles has already reached a length of almost 70 m. Packet forwarding may be necessary. Second, out-of-coverage or handover scenarios may lead to network partition. Again, packet forwarding may be necessary. Third, frequency reuse within the platoon is not possible resulting in a higher probability of packet collisions. The latter either requires the retransmission of the lost packets or compromises the safety of the platoon.

Uncoordinated packet forwarding, i.e., the retransmission of received packets according to a hop limit, leads to an explosive rise in the transmitted data quantity. The following simple example illustrates the importance of refraining from packet forwarding and highlights the low overheads achieved by *Feudalistic Platooning*: We assume that all platooned vehicles have access to both ad hoc and cellular networks. Furthermore, we neglect the transmission of any platoon management data: vehicles send CA messages only. As elaborated in Table 2, 768 kB of CAM data are sent in the presented simulation scenario. Each vehicle is responsible for 64 kB of CAM data. Sending this as redundant data over both ad hoc and C-V2X network results in 1536 kB of CAM data. Once the overall CAM data of a vehicle are retransmitted at least 18 times (18 × 64 kB = 1152 kB) over the duration of 300 s, the overall transferred data in the feudalistic platoon (2684 kB) are exceeded. Depending on the selected control policy and the length of the platoon, this may be quickly reached, for example, a platooned vehicle in [15] relies on data from the platoon leader and predecessor. If the CAM of a leader is forwarded by each vehicle, a platoon of 20 vehicles can be supported by the indicated data quantity (the head and tail of the platoon do not need to retransmit). With this approach, however, vehicles are only aware of their predecessor and the platoon leader. Lack of information about other platoon members may make platoon maneuvers more difficult. Similarly, the authors in [11] exchange vehicle control messages using 5G Mode-3, i.e., direct communication between the vehicles under network coverage. Furthermore, there, a complete picture of the composition of the platoon can only be obtained by packet forwarding.

The availability of information about other platoon members could be enhanced by sending the data also relying on cellular infrastructure. This would require an additional 768 kB of data, with the data volume sum already reaching 2304 kB. Included in this are the sending data to a central point, but not yet forwarding it to a specific vehicle and almost 86% of the data transmitted by the feudalistic platoon within the simulation scenario have now already been reached. Six forwarding transmissions of this kind (6 × 64 kB = 384 kB) already exceed the overall transferred data (2684 kB). The data volume increases significantly in accordance with Gauss summation, of course, when all the vehicles should become aware of each other.

Taking into account the observations described and the data relations expressed in Figure 8, Figure 9, Figure 10, Figure 11, Figure 12, Figure 13, Figure 14 and Figure 15, the key advantages of *Feudalistic Platooning* are clearly indicated:

1. Relieve networks:
  - Ad hoc network
    Communication that uses the ad hoc network is limited to one vassalage. On the one hand, a vassalage may agree on a specific communication channel that would ensure no interference for neighboring vassalages. On the other hand, transmission power may be reduced as broadcasted information is only of interest within the vassalage and is therefore short-ranged. This enables earlier frequency reuse.
  - LTE network
    Only one single vehicle per vassalage, namely the lord, accesses the cellular network using LTE communication. This decreases the communication overhead including the number of communication requests and simplifies the resource scheduling within the cellular network. This does not, however, reduce the net payload.
2. Improved operability:
  Operability does not depend on the quality of the network: once in coverage, the platoon may be updated downstream and upstream, e.g., about new platoon members. All platoon members are aware of each other. Out of coverage, this reduces transmissions by design to a bare minimum. Here, communication is limited to a single vassalage and C-V2X updates from the preceding vassalage. However, control policy should be selected appropriately: required data may come from the current or preceding vassalage.
3. Increased efficiency:
  Except for the knight of each vassalage, vehicles of single vassalages benefit from the short inter-vehicle distances, which increase platoon efficiency. Furthermore, negative influences from the surrounding traffic are limited as the vassalages are inseparable.
4. Improved coexistence:
  Large inter-vehicle distance between two vassalages reserve room for the surrounding traffic and unforeseeable maneuvers and create credit for emergencies.
5. Improved modularity:
  Vassalages may be built up on a situational basis and according to different indicators such as a common destination or driver availability. Furthermore, a quicker integration of entire vassalages into other platoons is enabled, instead of exiting and rejoining the individual vehicles.
6. Low overhead:
  Once *Feudalistic Platooning* is established, communication is purely data based and no additional management data are required.
7. Circumvent handover problem:
  Due to the fact that only one vehicle per vassalage is connected via LTE to the cellular network, the data dependency of different cells is not given. Whatever cell the lord is part of, its whole vassalage is also part of, even though technically they would already have switched cell.

### 5.4. Framing within Recent Selected Publications

Recent publications in the field of platooning focus on all layers: the vehicle layer, platoon layer, and transport layer. Table 3 lists their used communication technologies, mentions their key contributions, and highlights open questions. We emphasize that *Feudalistic Platooning* does not replace a single one of them, but its appropriate embedding addresses most of the open questions. These are explained as follows:

- The connection of all vehicles at any point in time: independent of the used communication technology, listed publications assume the connection of all vehicles at any point in time. This cannot be guaranteed under all circumstances and thus, the operability of the platoon is questionable. Especially when only cellular networks are used, handover scenarios lead to temporary connection losses. We explain in Section 5.3 how *Feudalistic Platooning* stays operable and circumvents handover problems.
- Strong data dependency: listed publications introduce strong data dependency, either assuming each vehicle is aware of every other vehicle or leader information is available throughout the platoon. This causes a large uplink and consequently also large downlink data traffic in cellular networks and drastically increases the ad hoc network load once single broadcasts are no longer sufficient. *Feudalistic Platooning* is able to steer message flow according to the needs of the vehicle controller. Critical data can be exchanged on a time-variable basis. Non-critical data are exchanged whenever possible (see Section 5.3).
- Network election: publications that consider the availability of both ad hoc and cellular networks assume that the data needed for the vehicle layer are exchanged using the ad hoc network. In contrast to that, data for upper layers are exchanged using the cellular network. However, the selection of the communication technology, e.g., on per-packet basis [12], would be possible at least for the vehicle layer and partly for the platoon layer. *Feudalistic Platooning* exploits the availability of multiple networks and adds redundancy on critical data. Data from higher layers can still be transmitted. Their timescale is increased anyways and thus, their criticality decreases [42].
- Heterogeneous platoons: vehicles within a platoon are heterogeneous by nature. Vehicles differ in type and in load. Thus, they offer different acceleration and deceleration capabilities. This needs to be considered by the vehicle layer [20]. In *Feudalistic Platooning*, vassalages can be used to group vehicles according to different indicators, e.g., according to their braking capability.
- Surrounding traffic and its variability: considering surrounding traffic is challenging. Increased inter-vehicle distance between two vassalages in a feudalistic platoon considers surrounding traffic already up-front. Variable vassalage size can counteract time-variable traffic density and its impact on the network quality (see Section 5.3).

## 6. Conclusions

We proposed *Feudalistic Platooning*, an extensive platooning concept that assigns roles to the vehicles of a platoon. According to these roles, vehicles reduce network load and steer communication flow within a platoon. Communication relies on two or more different networks. In the elaborated simulation scenario, we combined an ad hoc network (IEEE 802.11p) and cellular networks (LTE and C-V2X). We demonstrated that *Feudalistic Platooning* adds a low additional load only, while relieving each individual network. It adds redundancy on crucial platoon data, improves operability by ensuring operation outside of cellular coverage, and limits the impact of cellular handovers. Introducing vassalages, i.e., smaller groups of vehicles inseparable from the surrounding traffic, increases efficiency and modularity: vehicles are able to drive at small inter-vehicle distances and allowing the acceleration of platoon management maneuvers. The vassalage size impacts platoon efficiency, the network load as well as the road traffic. The use of smaller vassalages may be suitable in the event of heavy traffic volumes and high ad hoc network loads, since this would facilitate the coexistence with the surrounding traffic as well as shift the load to the cellular network. Heavy traffic volumes and good cellular network coverage can usually be found in and next to urban areas. In contrast, in situations of low traffic volume and in the event of weak cellular network coverage, e.g., in rural areas, the vassalage size should be increased, encouraging the use of the ad hoc network. Strong data relations are maintained within a vassalage: 5.8 times more data is transmitted compared to communication between two vassalages. Apart from the direct communication between two vassalages, LTE may be used to request data from other vassalages or platoons. These data are shared by one vehicle of the vassalage, stored and requested at/from a central server.

To make the most out of *Feudalistic Platooning*, the transport layer should also be considered if possible. Vehicles could be assigned to vassalages according to their destination or according to driver availability. Furthermore, a fleet operator may set up one or more vassalages that become part of a feudalistic platoon at least on one section of the route and otherwise operate as a normal platoon. A vassalage could be structured so as to consist of vehicles with similar characteristics, e.g., heavy load carriers. According to key characteristics, vassalages could be sorted within the feudal platoon. This might well be considered during splitting and merging maneuvers, for example, on freeway junctions. A sorted platoon may be advantageous in certain track segments, e.g., steep sections.

A limiting factor is that the presented conceptualization requires at least two specific types of networks: the first network needs to provide direct one-to-one or one-to-many communication. The second network needs to support communication with at least some vehicles that are distributed over the entire platoon with the result that vassalages can be established. For example, mobile networks cannot be considered for the first network. Similarly, VLC is not eligible for the second network. However, vassalage structure and role assignment may be adapted in accordance with the available communication technologies.

Further work remains to be conducted in simulating more sophisticated traffic scenarios, including that of the surrounding traffic as well as that in which the road characteristics vary. For these scenarios, appropriate parameters could be identified: transmission intervals, vassalage size, and inter-vehicle distance (inter-vassalage and intra-vassalage). Additionally, multi-cell and out-of-coverage scenarios, packet loss, and packet delay may be considered. Finally, different communication technologies may be used, e.g., visible light communication (VLC).

## Figures and Tables

**Table 1:** Simulation parameters used for the presented results.

Parameter	Value	Unit
Simulation Time	300	s
Merchant Interval	1	s
Ordering Interval	2	s
Shipping Interval	2	s
Constant Speed	30	m s^−1^
Platoon Length	12	number of vehicles
Vassalage Size	3	number of vehicles
Packet Reception Rate	100	%
Intra-Vassalage Vehicle Distance	8	m
Inter-Vassalage Vehicle Distance	70	m

**Table 2:** *Feudalistic Platooning*: transmission shares per node and type of communication. The transferred data volume refers to a period of 300 s. (*) Share values are rounded to two decimal places. Their sum results from the non-rounded values that may deviate slightly.

Type	Node	Role	Value	(*) Share Type	(*) Share Network	(*) Share Overall
			kB	%	%	%
**Overall**	n.a.	n.a.	2684	100.00	n.a.	100.00
	node[0]	Knight	128	4.77	n.a.	4.77
	node[1]	Lord	267	9.95	n.a.	9.95
	node[2]	Merchant	168	6.26	n.a.	6.26
	node[3]	Knight	208	7.75	n.a.	7.75
	node[4]	Lord	267	9.95	n.a.	9.95
	node[5]	Merchant	168	6.26	n.a.	6.26
	node[6]	Knight	208	7.75	n.a.	7.75
	node[7]	Lord	267	9.95	n.a.	9.95
	node[8]	Merchant	168	6.26	n.a.	6.26
	node[9]	Knight	208	7.75	n.a.	7.75
	node[10]	Lord	267	9.95	n.a.	9.95
	node[11]	Merchant	128	4.77	n.a.	4.77
	node[12]	Server	232	8.64	n.a.	8.64
	node[13]	Multicast	0	0.00	n.a.	0.00
	node[14]	Broadcast	0	0.00	n.a.	0.00
**IEEE 802.11p Overall**	n.a.	n.a.	768	100.00	100.00	28.61
*(CAM, DENM)*	node[0–11]	n.a.	64	8.33	8.33	2.38
**C-V2X Overall**	n.a.	n.a.	1584	100.00	100.00	59.02
C-V2X Multicast	n.a.	n.a.	768	100.00	48.48	28.61
*(CAM, DENM)*	node[0–11]	n.a.	64	8.33	4.04	2.38
C-V2X Intra Vassalage	n.a.	n.a.	696	100.00	43.94	25.93
	node[0]	Knight	0	0.00	0.00	0.00
	node[1]	Lord	114	16.38	7.20	4.25
	node[3]	Knight	80	11.49	5.05	2.98
	node[4]	Lord	114	16.38	7.20	4.25
	node[6]	Knight	80	11.49	5.05	2.98
	node[7]	Lord	114	16.38	7.20	4.25
	node[9]	Knight	80	11.49	5.05	2.98
	node[10]	Lord	114	16.38	7.20	4.25
C-V2X Inter Vassalage	n.a.	n.a.	120	100.00	7.58	4.47
	node[2]	Merchant	40	33.33	2.53	1.49
	node[5]	Merchant	40	33.33	2.53	1.49
	node[8]	Merchant	40	33.33	2.53	1.49
	node[11]	Merchant	0	0.00	0.00	0.00
**LTE Network Overall**	n.a.	n.a.	332	100.00	100.00	12.37
Ordering	n.a.	n.a.	16	100.00	4.82	0.60
	node[1]	Lord	4	25.00	1.20	0.15
	node[4]	Lord	4	25.00	1.20	0.15
	node[7]	Lord	4	25.00	1.20	0.15
	node[10]	Lord	4	25.00	1.20	0.15
Shipping	n.a.	n.a.	84	100.00	25.30	3.13
	node[1]	Lord	21	25.00	6.33	0.78
	node[4]	Lord	21	25.00	6.33	0.78
	node[7]	Lord	21	25.00	6.33	0.78
	node[10]	Lord	21	25.00	6.33	0.78
Ordering Response	n.a.	n.a.	232	100.00	69.88	8.64
	node[12]	Server	232	100.00	69.88	8.64

**Table 3:** Recent selected publications on different layers: used communication technologies, key contributions and open questions. The latter could be addressed by *Feudalistic Platooning*.

Publication	Communication	Key Contribution	Open Questions
COMPANION—Towards Co-Operative Platoon Management of Heavy-Duty Vehicles [43]. Year: 2015.	Hybrid network: (i) Cellular—3G for communication with central server; (ii) IEEE 802.11p for intra-platoon communication with extended ITS-G5 messages.	Comprehensive system that includes: (i) Strategic layer (i.e., transport layer); (ii) Tactical layer (i.e., platoon layer); (iii) Operational layer (i.e., vehicle).	(i) Connection loss and system operability; (ii) No communication flow: broadcast/multicast or retransmission required; (iii) Surrounding traffic.
A Predictive Framework for Dynamic Heavy-Duty Vehicle Platoon Coordination [42]. Year: 2019.	Single network: (i) Cellular—communication with central server.	Platoon coordination system on strategic layer within four-layered control architecture for platooning: (i) Service—good flows matched to vehicles and drivers; (ii) Strategic—match making (i.e., transport layer); (iii) Tactical—platoon management and maneuvers (i.e., platoon layer); (iv) Operational—vehicle controller (i.e., vehicle layer).	(i) Tactical and operational layers; (ii) Connection loss: out of coverage and handover; (iii) Surrounding traffic.
3GPP C-V2X and IEEE 802.11p for Vehicle-to-Vehicle communications in highway platooning scenarios [19]. Year: 2018.	Single network—one of the two networks: (i) C-V2X Mode-3 or Mode-4; (ii) IEEE 802.11p.	Vehicle Layer: (i) Investigate the suitability of IEEE 802.11p and C-V2X for platooning according to following performance metrics: inter-vehicle distance, message latency, and PRR; (ii) Results show that traffic density leads to congestion on the radio channel. Suitability for platooning in descending order—C-V2X Mode-3, C-V2X Mode-4, and IEEE 802.11p.	(i) Heterogeneous platoon; (ii) Cellular handover; (iii) No communication flow—broadcast/multicast or retransmission required; (iv) Time-varying traffic density.
Cloud-Assisted Distributed Control System Architecture for Platooning [44] Year: 2018	Single network: (i) Cellular—5G.	Three-layered distributed functional architecture to control and manage platoons assisted by cloud computing platform: (i) Trip planner (cloud); (ii) Road section manager (edge); (iii) Coordination control (vehicle).	(i) Connection loss: out of coverage and handover.
Platooning on the edge [45] Year: 2020	Single network: (i) Cellular—4G/5G.	Vehicle layer: (i) Predecessor-leader control of vehicle speed and acceleration is managed centralized according to the multi-access edge computing paradigm; (ii) Investigation of delay and packet loss in up- and downlink; (iii) Round trip message delays cannot be guaranteed below 150 ms—vehicle control must be performed on the vehicle.	(i) Other layers and their impact on the delay and packet loss; (ii) Connection loss: out of coverage and handover; (iii) Surrounding traffic; (iv) Varying data dependency.
Towards Edge Intelligence in the Automotive Scenario: A Discourse on Architecture for Database-Supported Autonomous Platooning [46] Year: 2022	Single network: (i) Vehicular dynamic spectrum access.	Vehicle layer: (i) Dynamic selection of the operating frequency for intra-platoon communication with the aim to minimize overall interference; (ii) Selection is supported by infrastructure (centralized, distributed, and hybrid); (iii) Unoccupied TV channels of the TV band are used for data offloading.	(i) Other layers and their impact; (ii) Connection loss and operability.

## Data Availability

The data presented in this study are openly available at https://github.com/rtobi/feudalistic_platooning_dataset. accessed on 3 June 2022.

## Abbreviations

The following abbreviations are used in this manuscript:

5G	Fifth-Generation Technology Standard for Broadband Cellular Networks
CAM	Cooperative Awareness Message
C-ITS	Cooperative Intelligent Transportation Systems
CPM	Collective Perception Message
CSMA/CA	Carrier Sense Multiple Access/Collision Avoidance
C-V2X	Cellular Vehicle to Everything
DENM	Decentralized Environmental Notification Message
HGV	Heavy Goods Vehicle
IEEE	Institute of Electrical and Electronics Engineers
LTE	Long-Term Evolution
PRR	Packet Reception Rate
SUMO	Simulation of Urban Mobility
V2X	Vehicle to Everything
VLC	Visible Light Communication
